# The Smart Therapy Model: a Fourth Wave framework for assessment-driven, integrative multimodal biopsychosocial psychotherapy

**DOI:** 10.3389/fpsyt.2026.1748511

**Published:** 2026-06-11

**Authors:** Alişan Burak Yaşar, Anıl Gündüz

**Affiliations:** 1 Department of Psychiatry, Faculty of Medicine, Istanbul Nişantaşı University, Istanbul, Türkiye; 2 Department of Clinical Psychology, İstanbul Kent Univeristy, İstanbul, Türkiye

**Keywords:** assessment, component analysis, dismantling studies, Fourth Wave, hypothesis, integrative psychotherapy, personalized medicine, psychotherapy

## Abstract

**Introduction:**

Despite decades of psychotherapy research yielding over 100 empirically supported treatment protocols, clinical practice continues to face persistent challenges, including high dropout rates averaging 15–20% in controlled settings and widespread treatment resistance. The central tension is the “research-practice gap”: whereas research validates singular, manualized protocols, clinicians predominantly operate eclectically. Critically, the field lacks a standardized framework to guide how clinicians should select and sequence interventions—not merely which protocols exist. Emerging paradigms such as the Research Domain Criteria (RDoC) and modular treatment approaches point toward individualized, mechanism-driven intervention; however, a unifying clinical integration framework remains absent.

**Hypothesis and theoretical framework:**

This paper proposes the “Smart Therapy” model as a theoretical framework and perspective on the foundations of a potential Fourth Wave of psychotherapy—conceptualized not as a novel philosophical school, but as an Integrated Multimodal Biopsychosocial Assessment-to-Intervention model. Grounded in the neuroscience of psychotherapy, the model posits that intervention selection should be driven by a standardized, three-domain assessment: (1) biological prerequisites; (2) deep neurostructural pattern issues (“Hardware”), referring to maladaptive memory traces encoded via trauma and adversity; and (3) “active cognitive-process habits (“Software”), referring primarily to maladaptive metacognitive regulatory styles centered on the Cognitive Attentional Syndrome (CAS)”.

**Model:**

The Smart Therapy model proposes a five-level, sequenced intervention dosing framework. Level 1 establishes collaborative goal-setting and value alignment (“The Compass”). Level 2 screens and addresses biological prerequisites—including neuroinflammation, hypothalamic-pituitary-adrenal (HPA) axis dysregulation, and micronutrient deficiencies—that constitute primary barriers to psychotherapeutic efficacy. Level 3 delivers the core psychological intervention, matched to the Hardware/Software assessment: “process-focused approaches, primarily Metacognitive Therapy (MCT), and where appropriate Acceptance and Commitment Therapy (ACT), for Software presentations. Pattern-reprocessing approaches (Eye Movement Desensitization and Reprocessing [EMDR], Schema Therapy, Psychodynamic Therapy) for Hardware presentations—the latter explicitly leveraging neuroplasticity mechanisms to restructure maladaptive memory networks. Levels 4 and 5 add behavioral activation and systemic interventions as augmentation strategies.

**Conclusion:**

Smart Therapy offers a framework to bridge the research-practice gap by standardizing the assessment process rather than the treatment protocol. Its central, falsifiable hypothesis is whether Hardware/Software assessment-guided intervention matching—particularly the use of MCT for Software-dominant presentations—may improve remission, reduce dropout, and increase treatment efficiency—compared to treatment-as-usual and single manualized protocols. Future research should prioritize component-based dismantling studies over monolithic protocol comparisons.

## 1 Introduction

The history of psychotherapy has been conceptualized as a succession of paradigmatic “waves” (1). The first wave established behaviorism as the foundation of psychological intervention; the second introduced cognitive mediation, exemplified by Beck’s Cognitive Therapy (2); and the third wave—encompassing approaches such as ACT, DBT, and Metacognitive Therapy (MCT)—re-emphasized contextual and process-level factors. Among these, MCT contributed a distinct model centered on metacognitive beliefs and the Cognitive Attentional Syndrome (CAS) (1, 3, 4). Each wave expanded the therapeutic toolbox. Yet in doing so, the field paradoxically fragmented: protocols multiplied, schools competed, and a widening gap emerged between the controlled world of research and the complex realities of clinical practice.

### 1.1 The research-practice gap

Even in the most rigorously controlled research settings—with carefully selected, relatively uncomplicated patient samples—psychotherapy dropout rates average 15–20% (5, 6) and vary dramatically by condition, from approximately 16% for PTSD to over 50% for personality disorders (7, 8). In real-world practice, where comorbidity is the norm and supervision the exception, these figures are assumed to be substantially higher (9). Despite more than 100 recognized evidence-based psychotherapy (EBP) schools (7), clinicians rarely adhere to manualized protocols. Instead, they adopt eclectic or integrative approaches, borrowing techniques across models in a manner that is clinically intuitive but methodologically unstandardized (3, 10). Critically, the type and “dose” of therapy a patient receives is determined more by the particular training background of the clinician than by any standardized assessment of the patient’s primary mechanism of pathology (11).

This is not a failure of individual clinicians—it reflects a structural gap in the field. Psychotherapy research is designed to validate singular protocols (e.g., a 12-session CBT manual), while clinical reality demands flexibility across comorbid, heterogeneous presentations. The result is a system in which neither research nor practice fully serves the patient.

### 1.2 Emerging frameworks: RDoC and modular approaches

Recent decades have generated important theoretical advances that gesture toward resolution. Insel and colleagues’ Research Domain Criteria (RDoC) framework represents a landmark shift, advocating for the dimensional, mechanism-based classification of psychopathology rooted in neuroscience—moving away from syndrome-based diagnoses toward transdiagnostic biological and psychological processes (12, 13). RDoC’s emphasis on identifying the *active mechanisms* of pathology rather than categorical diagnoses is directly aligned with the logic of the model proposed here.

Complementing this, the modular psychotherapy tradition has demonstrated the clinical and empirical utility of abandoning monolithic protocol structures in favor of flexible, individualized “modules” of intervention selected on the basis of the patient’s presenting problem profile (14–16). Modular approaches have shown advantages over standard protocols in real-world effectiveness, treatment engagement, and responsiveness to complex presentations (15).

However, both RDoC and modular approaches, for all their conceptual power, share a limitation in clinical translation: they provide *what* to target and *which* modules exist, but not a standardized, practitioner-accessible *process* for integrating biological and psychological assessment into a sequenced intervention framework. This integration gap is precisely what the Smart Therapy model seeks to address.

### 1.3 Component analysis and active ingredients

A further challenge is the field’s inadequate understanding of the active ingredients of change within any given therapy. Research conducted through component analysis and dismantling studies—methodologies designed to isolate the specific contributors to therapeutic efficacy—remains rare despite being critically important (17, 18). Even within CBT, which contains hundreds of distinct techniques, the relative contribution of cognitive versus behavioral components remains empirically unresolved (17). The Smart Therapy model is explicitly designed to be compatible with, and to accelerate, this line of inquiry: by framing intervention selection as mechanism-matched “dosing”, it renders the framework inherently testable at the component level.

### 1.4 The present proposal

This article presents the Smart Therapy model as a theoretical framework and perspective on a potential Fourth Wave of psychotherapy. It is proposed that this wave is not defined by a new singular philosophy of change. We propose that this wave is not defined by a new singular philosophy of change—as in prior major developments such as ACT, DBT, or MCT—but by a meta-level shift in clinical epistemology: a move from protocol-centered to assessment-centered practice. The Smart Therapy model operationalizes this shift through a five-level, standardized assessment-to-intervention framework, formally conceptualized as an *Integrated Multimodal Biopsychosocial Intervention*. In proposing this, it is recognized that this framework builds on—not replaces—the foundational contributions of RDoC, modular therapy, and three generations of evidence-based psychotherapy development. The aim is integration and clinical operationalization, not reinvention.

## 2 The Smart Therapy framework: an assessment-driven integrative model

### 2.1 Conceptual foundations

The Smart Therapy model goes substantially beyond unstructured clinical eclecticism. It proposes that psychotherapy be formally reconceptualized as an *Integrated Multimodal Biopsychosocial Intervention*—a structured clinical framework in which the selection, sequence, and intensity (“dose”) of interventions are determined by a standardized, multi-domain assessment of the primary mechanisms driving a given patient’s psychopathology.

This conceptual move rests on a foundational premise: that all effective psychotherapies, regardless of theoretical orientation, operate through shared neurobiological mechanisms. Therapy is, at its core, a method of facilitating experience-dependent change in the brain’s neural circuitry (19). This neurobiological grounding is not merely philosophical—it has direct clinical implications. If all effective therapies work through brain-level mechanisms, then the clinician’s primary task is not to choose between competing “schools,” but to identify *which mechanism of pathology is dominant* and to deliver the intervention that most precisely targets that mechanism. This is the logical core of the Smart Therapy clinical framework.

Furthermore, this framework posits that most psychotherapy research—by comparing monolithic protocols against one another (e.g., ACT vs. CBT)—systematically fails to identify the active ingredients of therapeutic change. Component analysis and dismantling studies remain the necessary but underfunded frontier of psychotherapy science (17, 18). The Smart Therapy model is explicitly designed to be compatible with this research agenda: by framing intervention as mechanism-matched “dosing,” it renders the framework testable at the component level.

### 2.2 The two-domain psychological assessment: hardware and software

The central and distinguishing conceptual heuristic of the Smart Therapy framework is a two-domain model of psychological pathology. Following a biological screening (addressed in Level 2 of Section 3), the clinician conducts a structured assessment across two orthogonal but frequently co-occurring psychological dimensions: *Hardware* and *Software*. These terms are used as clinical heuristics—not as reductive analogies—to help clinicians and, where appropriate, patients, organize the nature of the primary psychological problem. These domains are not mutually exclusive; most clinical presentations involve elements of both, and the clinician’s task is to assess the *relative intensity and priority* of each driver to determine the sequence and weighting of interventions.

#### 2.2.1 Hardware: deep neurostructural pattern issues

The term “Hardware,” in this framework, does not refer to fixed, immutable biological substrate— a misreading this paper explicitly wishes to forestall. Rather, it refers to psychopathology that is *deeply encoded*: maladaptive patterns laid down by traumatic experience, chronic adversity, or formative relational failures, which have become structurally consolidated within long-term memory networks and associated neural circuits (20–22).

The neuroscience of traumatic memory provides the foundational rationale for this distinction. Traumatic and adverse experiences are encoded through stress-potentiated synaptic consolidation, involving the amygdala, hippocampus, and prefrontal cortex in ways that differ qualitatively from the encoding of ordinary autobiographical memory (22, 23). The resulting memory traces are not simply “thoughts about the past”—they are somatically encoded, affect-laden patterns that actively organize the individual’s perception of self, others, and threat in the present. In clinical terms, this is what is meant by “Hardware”: the problem is not the content of a belief that can be logically examined and disputed, but a deeply consolidated, subcortically mediated experiential pattern that must be *accessed, processed, and reconsolidated*—not merely reasoned about.

Crucially—and this point is central to the clinical and theoretical coherence of this framework—**Hardware is not fixed**. The neuroscience of memory reconsolidation has demonstrated that even well-consolidated long-term memories, when reactivated, re-enter a labile state during which they are susceptible to modification before restabilization (24, 25). This process—*memory reconsolidation*—is now understood to be a primary neurobiological mechanism through which trauma-focused psychotherapies such as Eye Movement Desensitization and Reprocessing (EMDR) (22), Schema Therapy (26), Psychodynamic Therapy, and Transference-Focused Psychotherapy (TFP) exert their effects (20). Through repeated cycles of activation and reprocessing within a safe relational and therapeutic context, these approaches literally *rewire* the neural networks that encode maladaptive patterns—a process supported by the well-established principle of experience-dependent neuroplasticity (19, 27). The “hard drive” metaphor, therefore, is not one of pessimism or determinism—it is one of *depth*. These patterns require a qualitatively different therapeutic approach precisely *because* they are deeply encoded; not because they are beyond change, but because superficial cognitive interventions cannot reliably access and reconsolidate subcortically mediated memory networks.

Hardware presentations are clinically identified through validated instruments including the Adverse Childhood Experiences (ACE) questionnaire, the PTSD Checklist (PCL-5), and structured personality assessment tools such as the SCID-II. They are characterized by affect dysregulation, identity instability, relational dysfunction, somatic symptoms, and the presence of intrusive or dissociative phenomena. A frequent clinical concern is that exploration of historical material may increase pathological rumination; however, current evidence does not support this concern for competently applied, appropriately paced trauma- and pattern-focused therapies (28).

#### 2.2.2 Software: active cognitive-process habits and metacognitive dysregulation

Where Hardware refers to the *content and structure* of encoded experiential patterns, Software refers to the *process and style* of ongoing cognitive activity. The Software dimension conceptualizes psychopathology as a dysfunction of the individual’s cognitive “operating system”—specifically, the metacognitive regulatory processes that govern how the individual relates to, and manages, their own mental activity (21, 29).

The most clinically precise articulation of this construct is Adrian Wells’ Metacognitive Model and its associated concept of the Cognitive Attentional Syndrome (CAS)—.a constellation of sustained worry, rumination, threat monitoring, and maladaptive cognitive-attentional coping strategies driven by dysfunctional metacognitive beliefs about the controllability, meaning, and necessity of repetitive thinking (21, 29, 30). Critically, in the Software model, the primary pathogen is not the *content* of the patient’s thoughts—it is the *process*: the rigid, perseverative engagement with thought that the patient’s metacognitive beliefs both trigger and maintain. This distinction has profound implications for treatment selection. In CAS-dominant presentations, repeated content-focused engagement with thoughts may be insufficient and may, in some cases, prolong perseverative processing by maintaining the sense that thoughts require analysis. What is required instead is an intervention that targets the processing *mode* itself (21, 29).

Metacognitive Therapy (MCT) was developed precisely for this purpose, targeting the metacognitive beliefs that initiate and maintain the CAS (21, 29, 30). Metacognitive Therapy (MCT) is the primary intervention for Software presentations because it directly targets the metacognitive beliefs and regulatory processes that initiate and maintain the CAS. Acceptance and Commitment Therapy (ACT) may serve as a complementary process-focused approach, particularly through cognitive defusion and changes in the individual’s relationship to thought (31, 32). Both approaches modify the underlying processing rules rather than rewriting individual cognitive content—constituting what the Smart Therapy framework terms “patching the operating system.” A growing body of evidence supports the efficacy of these process-focused interventions across anxiety, depression, and PTSD presentations (33).

Software presentations are identified through validated tools including the MCQ-30 and CAS-1, and are characterized by perseverative thinking, difficulty disengaging attention, beliefs about the uncontrollability or usefulness of thinking, and recurrent thought-control efforts that paradoxically maintain distress. They are clinically characterized by predominantly process-level symptoms: perseverative thinking, difficulty disengaging from rumination, future-directed worry, and the phenomenological sense that the patient’s mind “will not stop”—in the absence of the affective dysregulation or relational disturbance characteristic of Hardware presentations.

#### 2.2.3 The heterogeneous presentation: co-occurring hardware and software

In clinical practice, pure Hardware or pure Software presentations are the exception rather than the norm. The majority of patients present with significant elements of both: a history of adversity that has generated maladaptive relational patterns (Hardware), compounded by secondary metacognitive dysregulation through which the patient ruminates chronically about that history or worries catastrophically about future threats (Software). The Smart Therapy framework does not impose an artificial binary on this complexity. Rather, it asks the clinician to conduct a dimensional assessment of the *relative intensity and clinical priority* of each domain and to design an intervention sequence accordingly. This assessment-to-dosing logic is elaborated fully in the Five-Level Clinical Process Model described in Section 3.

## 3 A five-level intervention dosing framework: the Smart Therapy clinical process model

The following framework represents the clinical operationalization of the Smart Therapy model. It is organized as a sequenced, five-level intervention structure in which the levels represent clinical *priorities* and *domains of assessment and intervention* that inform one another iteratively across the course of treatment. The term “dose” is used throughout not to imply patient passivity or a purely medical model, but to highlight the need for *precision* in intervention selection: moving beyond the intuitive but unstandardized eclecticism that currently characterizes clinical practice (10), toward a deliberate, assessment-informed prescription of therapeutic components.

### 3.1 Level 1: collaborative goal-setting and value alignment — “The Compass”

The first level of the Smart Therapy framework establishes the navigational foundation for all subsequent intervention. Before any assessment of biological status, Hardware patterns, or Software processes can meaningfully inform treatment, the clinician and patient must establish a shared, explicit understanding of *why* therapy is being undertaken and *toward what end*. This is not a preliminary formality—it is a therapeutic intervention in its own right with direct empirical support for its impact on outcomes.

Meta-analytic evidence demonstrates that goal consensus and therapeutic collaboration are among the most robust predictors of psychotherapy outcome, independent of modality (34, 35). Collaborative goal-setting strengthens the therapeutic alliance, which itself is one of the strongest and most replicated predictors of therapeutic success across all orientations (36). Critically, the goal-setting process at this level is not clinician-directed. Drawing on third-wave and solution-focused approaches, the clinician’s role is to collaboratively elicit the patient’s *preferred future* (37) and *core life values* (38, 39), and to translate these into concrete, personalized, and measurable intermediate objectives (40, 41).

Goals established at Level 1 perform several functions simultaneously within the Smart Therapy framework. They provide motivational scaffolding that sustains engagement during the more demanding work of subsequent levels. They establish a measurable baseline against which progress can be tracked. And they ensure that the subsequent assessment process—biological screening, Hardware/Software evaluation—is oriented toward outcomes that are meaningful to the patient rather than predetermined by the clinician’s theoretical preferences (42, 43). Level 1 is the “compass” that ensures all subsequent navigation serves the patient’s own direction of travel.

### 3.2 Level 2: biological prerequisites — preparing the neurological ground for psychotherapy

Once the therapeutic compass is established, the Smart Therapy framework directs clinical attention to the biological domain. This level does not constitute a psychotherapeutic intervention in itself; rather, it represents a prerequisite assessment and stabilization phase that determines the *capacity* of the patient’s neural system to benefit from psychological intervention. This is among the most clinically important and systematically underutilized components of current psychiatric practice.

The theoretical rationale is grounded in neuroscience: psychotherapy works through the brain (19). Cognitive reappraisal, memory reconsolidation, behavioral activation, and metacognitive modification are all neurobiological processes. Any biological factor that impairs synaptic plasticity, prefrontal regulatory capacity, or stress system regulation will, by definition, attenuate the efficacy of psychological intervention. Biological stabilization, in this framework, is the equivalent of preparing the soil before planting: without adequate neurological substrate, even the highest-quality psychological intervention cannot achieve its potential effect.

#### 3.2.1 Level 2.1: pharmacological stabilization

For presentations marked by acute biological dysregulation or impaired capacity to engage in psychological work, pharmacological stabilization may be required before or alongside psychotherapy. In such cases, psychotherapy is typically introduced as an adjunctive and complementary intervention once sufficient stability has been established (44, 45). In these presentations, psychotherapy functions as an essential adjunct intervention. The Smart Therapy framework explicitly incorporates this clinical reality rather than positioning psychological intervention as invariably primary.

#### 3.2.2 Level 2.2: neuroinflammatory and neuro-metabolic factors

A growing and convergent body of evidence demonstrates that chronic low-grade neuroinflammation represents a significant and underappreciated barrier to psychotherapy efficacy. This level therefore mandates routine screening for modifiable neuro-metabolic and inflammatory factors, particularly in treatment-resistant presentations (44, 48).

Elevated inflammatory markers—CRP, interleukin-6 (IL-6), and tumor necrosis factor-alpha (TNF-α)—have been consistently associated with reduced response to both pharmacological and psychological treatments for depression and anxiety (49, 50). Mechanistically, neuroinflammation impairs synaptic plasticity by interfering with BDNF signaling and suppressing hippocampal neurogenesis—the neurobiological substrate of learning, memory reconsolidation, and cognitive flexibility (49). This has a direct implication for the Smart Therapy model: a patient presenting with elevated inflammatory markers may demonstrate attenuated response to metacognitive (Software) intervention not because the intervention is inappropriate, but because the neurobiological machinery required for cognitive change is itself compromised.

HPA axis dysregulation, indexed by chronically elevated cortisol, has similarly been shown to predict poorer response to psychotherapy for depression (51), and to impair the top-down prefrontal emotional regulatory capacity that most psychotherapeutic interventions seek to strengthen (52). Insulin resistance and mitochondrial dysfunction have been independently associated with depressive and anxiety symptoms through their effects on dopaminergic signaling and cellular energy metabolism (53–55)—factors that may require metabolic intervention before meaningful psychological gains are achievable.

#### 3.2.3 Level 2.3: micronutrient deficiencies and hormonal imbalances

Common and easily correctable micronutrient deficiencies represent a clinically underappreciated confound in the assessment of treatment resistance. Vitamin D deficiency—highly prevalent in populations with mood and anxiety disorders and routinely unscreened—has been shown to impair serotonergic and dopaminergic neurotransmission, reduce neuroprotection, and increase inflammatory burden (50), all of which attenuate the synaptic plasticity mechanisms through which psychological intervention operates. Similarly, B12 deficiency, magnesium insufficiency, and thyroid dysfunction are each capable of producing or exacerbating psychiatric symptom profiles that may mimic or compound primary psychopathology (46, 47, 56, 57).

The clinical logic is explicit and actionable: if a patient’s apparent “treatment resistance” to a Software intervention (e.g., MCT for rumination) is partially attributable to Vitamin D deficiency-mediated impairment of synaptic plasticity, the appropriate response is not to escalate pharmacotherapy or label the patient as a non-responder—it is to correct the deficiency and reassess. This does not invalidate the Software formulation; rather, it suggests that an otherwise appropriate MCT intervention may appear less effective until the relevant biological barrier is corrected.

#### 3.2.4 Level 2.4: genetic, epigenetic, and technology-augmented assessment — a future horizon

This subsection is presented explicitly as a future research trajectory rather than current clinical practice. Epigenetic modifications—particularly DNA methylation patterns in genes governing immune function and stress response—are increasingly recognized as mechanistic mediators between early adversity and adult psychopathology, including PTSD (58–62). While individual epigenomic profiling is not yet a viable clinical tool for individual treatment selection, the direction of travel is clear. AI-augmented assessment tools capable of analyzing multimodal biological and behavioral data—from cortisol dynamics to voice acoustic markers of stress—may substantially enhance the precision of biological-to-psychological matching in future iterations of this framework (58). This boundary is acknowledged clearly: the clinical recommendations of Level 2.1–2.3 are grounded in current evidence and immediately implementable; Level 2.4 represents the horizon toward which the model points.

### 3.3 Level 3: core psychological intervention — dosing hardware and software

Level 3 constitutes the primary psychological “dose” of the Smart Therapy framework, directly informed by the Hardware/Software assessment. The clinician selects an intervention not because it is their preferred modality, but because it is *mechanistically matched* to the patient’s primary domain of pathology.

#### 3.3.1 Level 3.1: dosing for software — targeting the metacognitive operating system

When the pre-treatment assessment identifies the Cognitive Attentional Syndrome (CAS) as the primary driver—indexed by elevated MCQ-30 scores, high rumination and worry frequency, and when CAS is the primary maintaining mechanism—indexed by elevated MCQ-30 scores, high rumination and worry frequency, and no dominant trauma-network activation or severe personality pathology interfering with process-focused work—the intervention dose consists of process-focused techniques designed to modify the patient’s metacognitive regulatory style rather than the content of their thoughts (21, 29).

Metacognitive Therapy (MCT) is the primary evidence-based intervention for this presentation (21, 30). MCT targets the positive metacognitive beliefs that initiate the CAS (“Ruminating helps me understand my problems”) and the negative metacognitive beliefs that maintain it (“My worry is uncontrollable and dangerous”), alongside the attentional and behavioral strategies that sustain the syndrome. A growing body of randomized controlled trial evidence supports MCT’s efficacy across anxiety, depression, and PTSD presentations (33). ACT techniques, particularly cognitive defusion, offer a complementary approach: rather than modifying metacognitive beliefs directly, defusion techniques alter the individual’s *relationship* to thought, reducing the “stickiness” of ruminative content without requiring direct engagement with it (31, 32). Both approaches modify the underlying processing rules rather than individual cognitive content—constituting what the Smart Therapy framework terms “patching the operating system.” In practice, this may involve techniques such as Attention Training Technique, Detached Mindfulness, and structured postponement of worry or rumination, all aimed at reducing CAS activation and strengthening flexible attentional control.

This is consistent with the empirical observation that second-wave (CBT) practitioners emphasize cognitive restructuring—a content-focused intervention—while third-wave practitioners emphasize process-level change strategies (3). The Smart Therapy model proposes that this difference in clinical emphasis reflects an implicit assessment of whether the patient’s primary problem is one of *content* (maladaptive beliefs requiring restructuring) or *process* (dysfunctional metacognitive regulation requiring mode-shifting). Making this assessment explicit and standardized is among the core contributions of the framework. The distinctive contribution of MCT within this framework is that it makes this process-level assessment explicit by targeting metacognitive beliefs, attentional deployment, and perseverative thinking styles rather than primarily evaluating thought content.

#### 3.3.2 Level 3.2: dosing for hardware — rewiring through memory reconsolidation

When the assessment identifies Hardware as the primary driver—significant trauma history, adverse childhood experiences, personality structure pathology, or relational pattern disturbance—the intervention dose must consist of approaches designed to access, activate, and reconsolidate the deeply encoded neurostructural patterns that constitute the pathology (20, 22).

The neurobiological rationale is grounded in memory reconsolidation research: reactivated traumatic memories, brought back into a labile state through careful therapeutic technique, can be modified before restabilization—reducing the affective charge and maladaptive associative structure of the original memory trace (21, 29). EMDR, whose mechanisms include dual-attention stimulation-facilitated memory reprocessing and reconsolidation (22), has an extensive meta-analytic evidence base for trauma-related presentations and is recommended as a first-line treatment for PTSD by the WHO, NICE, and the American Psychological Association. Schema Therapy targets the early maladaptive schemas—deep structural patterns of self and world—that constitute the cognitive-affective Hardware of personality disorder presentations (26). Psychodynamic approaches and Transference-Focused Psychotherapy (TFP) access the relational Hardware through the therapeutic relationship itself, using transference phenomena as both data and vehicle for pattern-level change (20).

Through repeated cycles of activation and reprocessing within a safe relational and therapeutic context, these approaches literally *rewire* the neural networks that encode maladaptive patterns—a process supported by the well-established principle of experience-dependent neuroplasticity (19, 27). As emphasized in Section 2.2.1, the “Hardware” designation communicates *depth of encoding*, not intractability. Current evidence does not support the clinical concern that competently applied, appropriately paced trauma- and pattern-focused therapies increase pathological rumination (28); processing Hardware is fundamentally distinct from unguided ruminative dwelling.

#### 3.3.3 Level 3.3: mixed presentations — sequential and combinatorial dosing

For the majority of patients, who present with clinically significant Hardware and Software components, the framework requires the clinician to assess the relative dominance and sequencing priority of each domain. As a general clinical heuristic, when active neurobiological dysregulation—fear network activation, dissociation, somatization—is prominent, Hardware-focused intervention typically takes priority: unprocessed trauma will continue to feed Software-level rumination regardless of metacognitive intervention. When perseverative thinking, threat monitoring, and attentional inflexibility are sufficiently intense to interfere with trauma processing, an initial MCT phase may be indicated to reduce CAS activation and improve readiness for Hardware-focused intervention. The Smart Therapy model does not prescribe a single sequence—it prescribes a *sequencing logic* grounded in mechanism-based assessment. The relative weighting of Hardware versus Software at Level 3 is directly informed by the profile established in the pre-treatment assessment phase.

### 3.4 Level 4: behavioral activation — augmenting the core dose

Level 4 provides a structured behavioral augmentation to the core psychological intervention. Behavioral Activation (BA)—the systematic scheduling and reinforcement of engagement with rewarding and value-consistent activities—has demonstrated efficacy comparable to full CBT in the treatment of depression, with substantially greater simplicity and accessibility (63). Structured exercise represents an additional, powerfully evidence-based augmentation strategy: a recent network meta-analysis of randomized controlled trials demonstrated that exercise produces moderate-to-large effect sizes for depression outcomes, exceeding psychotherapy alone in several comparisons (64), with additional evidence for reductions in suicidal ideation when combined with CBT (65). Within the Smart Therapy framework, Level 4 interventions act through behavioral and neurobiological mechanisms—including BDNF upregulation through aerobic exercise—that are complementary to and synergistic with the psychological work of Level 3.

### 3.5 Level 5: systemic intervention — addressing the relational environment

The final level of the Smart Therapy framework acknowledges a principle well-established in systems theory but frequently neglected in individually oriented clinical practice: the individual cannot be treated in isolation from the relational systems that maintain, modify, or exacerbate their psychopathology (66). When individual-level intervention has reached a plateau—or when functional assessment reveals that the patient’s presenting problems are substantially maintained by dysfunctional family, couple, or social system dynamics—the integration of systemic intervention is both clinically logical and empirically supported.

Meta-analytic evidence supports the efficacy of systemic therapy for adult depression (67), and for OCD maintained by family accommodation patterns (68). A comprehensive meta-analytic review of 38 randomized controlled trials demonstrated the efficacy of systemic approaches across a range of adult presentations (69). Couple and family therapy, delivered as an adjunct to or integration with individual work, can produce rapid and durable changes that individual intervention alone cannot achieve when relational system factors are primary maintainers of pathology (68, 70, 71). Level 5 thus completes the Smart Therapy model’s biopsychosocial scope: from the neurochemical (Level 2) to the relational and systemic (Level 5), the framework holds the full complexity of the human person in clinical view.

## 4 Discussion and future directions

### 4.1 The Fourth Wave as a meta-level epistemological shift

The claim that Smart Therapy constitutes a theoretical foundation for a potential “Fourth Wave” of psychotherapy merits careful justification, and it is offered with appropriate epistemic humility. Previous waves of psychotherapy were defined by the introduction of new philosophical frameworks for understanding psychological change: behaviorism, cognitive mediation, and contextual acceptance-based processes, respectively (1). Each wave generated new techniques, new vocabularies, and new schools—and in doing so, inadvertently contributed to the fragmentation that now characterizes the field. The research-practice gap (10) is not merely a failure of dissemination; it is a structural consequence of a field organized around competing philosophical traditions rather than shared mechanisms.

The Fourth Wave proposed here is therefore not another technique not another school, and not another philosophical orientation. It is a *meta-level shift in clinical epistemology*: a move from protocol-centered to assessment-centered practice. Where previous waves asked “which therapy is best?”, the Fourth Wave asks “which mechanism is primary in this patient, and which intervention most precisely targets that mechanism?” This reframing does not discard the accumulated evidence base of three generations of psychotherapy research—it reorganizes it. ACT, EMDR, MCT, Schema Therapy, Behavioral Activation, and systemic intervention are not competing alternatives within this framework; they are components of a shared toolbox, selected and sequenced according to the logic of mechanism-matched dosing.

Critically, it is acknowledged that this vision builds directly upon—not against—the landmark contributions of the RDoC framework (12, 13) and the modular psychotherapy tradition (14–16). RDoC established the principled case for mechanism-based rather than syndrome-based approaches to psychopathology; the modular tradition demonstrated the clinical and empirical feasibility of flexible, assessment-guided intervention over manualized protocols. The Smart Therapy model’s contribution is to operationalize this logic into a practitioner-accessible, five-level clinical process framework—bridging the gap between high-level theoretical principles and the concrete, session-by-session decisions that clinicians must make daily.

This article also addresses the tension between the “dosing” metaphor (which may connote a passive, medical model of the patient) and the collaborative, client-centered ethos foundational to Level 1 of the framework. The term “dosing” is used deliberately, but not uncritically: it is intended to capture the *prescriptive precision* of intervention selection, not to imply patient passivity. The collaborative compass of Level 1 ensures that all subsequent clinical decisions remain anchored in the patient’s own values and goals.

### 4.2 Bridging the research-practice gap: standardizing the assessment, not the protocol

The Smart Therapy framework addresses the research-practice gap through a conceptual inversion that is regarded as its most practically significant contribution. Current attempts to bridge this gap largely focus on improving clinician adherence to manualized protocols—a strategy that has achieved limited success because it fails to resolve the fundamental mismatch between the standardization demands of research and the heterogeneity demands of practice (10). The Smart Therapy model proposes a different solution: rather than standardizing the *protocol*, standardize the *assessment*.

A standardized protocol imposes a predetermined sequence of techniques on a heterogeneous patient population, treating variability as noise to be controlled. A standardized assessment, by contrast, systematically maps that variability—biological, Hardware, Software—and uses it as the primary input for individualized intervention selection. The protocol becomes a consequence of the assessment rather than its replacement. This is compatible with the evidence base: randomized controlled trials of manualized treatments can be understood, within this framework, not as evidence that all patients should receive a given protocol, but as evidence that the active components of that protocol are efficacious for patients whose primary mechanism of pathology aligns with the protocol’s target.

This reframing also offers a resolution to the longstanding “equivalence paradox” in psychotherapy research, in which diverse therapies produce similar aggregate outcomes across unselected patient populations (36). From the Smart Therapy perspective, equivalence at the aggregate level is entirely expected when patients are not stratified by mechanism: the averaging of patients for whom a given therapy is well-matched with those for whom it is poorly matched will naturally attenuate between-condition differences. Mechanism-matched dosing predicts that stratified trials will reveal substantially larger and more clinically meaningful between-condition differences than the existing literature has been able to detect.

### 4.3 Testable hypotheses and falsifiability

The Smart Therapy model, taken as a whole, is too complex and multidimensional to constitute a single hypothesis testable in a single trial. Its falsifiability lies in its mechanistically specific, testable components. Therefore, a hierarchy of four hypotheses is proposed, progressing from the most specific to the most integrative.

**Primary hypothesis — level 3 mechanism matching:** Patients assessed as presenting with a primary Software profile (elevated CAS/MCQ-30 scores, low ACE/PCL-5 scores) and allocated to receive a Software-matched intervention (MCT or ACT) will demonstrate significantly faster remission, lower dropout rates, and greater efficiency of therapeutic gain—measured in sessions to response—compared to patients with equivalent Software profiles receiving a Hardware-matched intervention (EMDR or Schema Therapy), and compared to treatment-as-usual. The reciprocal hypothesis applies to Hardware-primary presentations. This is the core falsifiable claim of the Smart Therapy framework: that mechanism-matched dosing outperforms unmatched active treatment and single manualized protocols on clinically meaningful outcomes (17, 18).

**Secondary hypothesis — level 2 biological priming:** Patients with identified and corrected biological barriers (e.g., Vitamin D deficiency, elevated inflammatory markers) at Level 2 will demonstrate significantly greater response to subsequent psychological intervention at Level 3, compared to patients with equivalent biological findings who did not receive Level 2 correction prior to psychological treatment (49–51). This hypothesis tests the “preparing the neurological ground” logic central to the model’s biopsychosocial integration.

**Tertiary hypothesis — assessment reliability:** The two-phase psychological assessment (Hardware/Software) using standardized instruments—MCQ-30, ACE, PCL-5—will demonstrate acceptable inter-rater reliability, test-retest stability, and predictive validity for treatment matching decisions. Without demonstrated assessment reliability, the dosing logic cannot be meaningfully operationalized.

**Quaternary hypothesis — integrative framework superiority:** Patients treated according to the full five-level Smart Therapy protocol—incorporating collaborative goal-setting, biological screening, mechanism-matched psychological intervention, behavioral augmentation, and systemic intervention where indicated—will demonstrate superior long-term outcomes, lower relapse rates, and higher treatment completion than patients receiving single manualized protocols or treatment-as-usual across a transdiagnostic sample.

These hypotheses are, in principle, testable within the existing methodological infrastructure of psychotherapy research. What they require is a shift in research design priorities: from monolithic protocol comparisons toward stratified, component-based dismantling studies (17, 18) that treat the mechanism-matching hypothesis as the primary experimental variable.

Future studies should specifically investigate: whether the two-phase Hardware/Software assessment can be reliably operationalized into a brief, validated intake battery; what the specific moderators and mediators of matching efficacy are; how intervention sequencing should be determined for mixed Hardware/Software presentations; what the quantifiable impact of Level 2 biological stabilization is on Level 3 effect sizes; and how AI-augmented assessment tools can be validated for clinical deployment without introducing opacity and accountability problems (58).

### 4.4 The horizon: AI-augmented precision dosing and the future of the framework

This paper closes with a forward-looking proposition intended neither as hyperbole nor as distraction from the framework’s present, immediately implementable clinical logic, but as a statement of the research trajectory toward which the Smart Therapy model naturally points.

The fundamental challenge of mechanism-matched dosing is informational: the clinician must assess multiple biological, psychological, and contextual dimensions simultaneously, weight them appropriately against a rapidly growing evidence base, and translate that assessment into a precise, individualized intervention sequence. The current implementation of the Smart Therapy framework—using validated questionnaires, structured clinical interviews, and biological laboratory panels—represents the best currently available approximation of this ideal.

The medium-term future, however, offers substantially more powerful tools. Advances in machine learning applied to multimodal biological and behavioral data—integrating inflammatory biomarker profiles, genetic and epigenetic screening, vocal and linguistic markers of cognitive processing style, wearable physiological monitoring, and treatment response trajectories—may eventually support AI-augmented clinical decision systems capable of generating individualized, mechanism-matched dosing recommendations with a precision that exceeds unaided clinical judgment (58). Early work in computational psychiatry has already demonstrated proof-of-concept for biomarker-informed treatment prediction in depression (72) and for machine learning-based identification of trauma-related epigenetic signatures (60).

Critically, this is not envisioned as the replacement of clinical judgment by algorithmic prescription. The collaborative, relational, and ethically responsive dimensions of psychotherapy—embodied most directly in Level 1 of the Smart Therapy framework—are not reducible to data and are not appropriately delegated to algorithmic systems. Rather, AI-augmented assessment is envisioned as a powerful augmentation of the clinician’s informational bandwidth, while leaving the relational and ethical dimensions of clinical decision-making firmly in human hands.

The Smart Therapy model is not a finished edifice—it is a theoretical foundation and a research program. Its contribution, if any, lies not in resolving the complexities of personalized psychotherapy but in providing a structured, falsifiable, and clinically actionable framework within which those complexities can be more rigorously explored. By standardizing the assessment rather than the protocol, it aims to bring the precision of evidence-based medicine to bear on the irreducible individuality of human psychological suffering—without sacrificing the humanity that makes psychotherapy, at its best, genuinely transformative.

## Data Availability

The original contributions presented in the study are included in the article/supplementary material. Further inquiries can be directed to the corresponding author.

## Appendix: The Smart Therapy Preliminary Clinical Assessment Tool (ST-PCAT)

To facilitate the practical application and future empirical investigation of the Smart Therapy model, the following preliminary assessment checklist is provided. This tool is designed to assist clinicians in rapidly orienting toward the primary domain of psychopathology (Hardware vs. Software) to guide initial “dosing” decisions.

**Phase 1: Biological Prerequisite Screening (Level 2).**

• [] Does the patient present with acute biological dysregulation requiring immediate pharmacological stabilization (e.g., active psychosis, acute mania)?

• [] Are there unaddressed metabolic, hormonal, or nutritional confounds (e.g., severe Vitamin D deficiency, thyroid dysfunction, known systemic inflammation)?

• Clinical Action: If yes, initiate Level 2 biological stabilization concurrently or prior to Level 3 psychological intervention.

**Phase 2: Hardware vs. Software Differential Assessment (Level 3)** *Domain A: “Software” Indicators (Process/Metacognitive Focus)*

• [] Is the patient’s primary distress maintained by perseverative thinking (rumination about the past or worry about the future)?

• [] Does the patient express beliefs about the uncontrollability or necessity of their own thinking process (e.g., “My mind won’t stop,” “I have to figure this out”)?

• [] Is the presentation largely free from severe identity instability, dissociative symptoms, or active trauma re-experiencing?

*Clinical Action:* Domain A is dominant, select process-focused interventions, primarily MCT, with ACT as a possible complementary option depending on the case formulation.

• Domain B: “Hardware” Indicators (Pattern/Structural Focus)

• [] Is there a significant history of adverse childhood experiences or relational trauma driving current symptoms?

• [] Does the patient exhibit affect dysregulation, somatization, or rigid, deeply encoded maladaptive interpersonal patterns?

• [] Are core pathogenic beliefs inextricably linked to specific, emotionally charged historical memory networks?

• Clinical Action: If Domain B is dominant, select pattern-reprocessing interventions (e.g., EMDR, Schema Therapy, Psychodynamic Therapy).

**Integration of Behavioral and Systemic Augmentation (Levels 4 & 5)**

Regardless of the primary domain assessment (Domain A or B), clinicians should proactively plan for the integration of behavioral and systemic interventions at each stage of the treatment process. Behavioral activation and exercise should be considered to augment core psychological doses, while systemic assessment remains essential to identify relational factors that may maintain or exacerbate individual pathology. The Smart Therapy framework encourages the iterative incorporation of these levels to ensure a comprehensive, biopsychosocial approach throughout the course of care.
